# Effects of *Yiqi Huoxue* Decoction on Post-Myocardial Infarction Cardiac Nerve Remodeling and Cardiomyocyte Hypertrophy in Rats

**DOI:** 10.1155/2021/5168574

**Published:** 2021-08-21

**Authors:** Hui Wang, Yuqin Zhang, Shuwen Guo, Jiani Wu, Wang'ou Lin, Binyue Zhang, Pengfei Feng, Lulu Wei, Yunke Liu, Jie Chen, Yufei Li

**Affiliations:** ^1^School of Traditional Chinese Medicine, Beijing University of Chinese Medicine, Beijing 100029, China; ^2^School of Acupuncture-Moxibustion and Tuina, Beijing University of Chinese Medicine, Beijing 100029, China; ^3^Fangshan Hospital Beijing University of Chinese Medicine, Beijing 102499, China; ^4^Wang Jing Hospital of CACMS, Beijing 100102, China; ^5^Yantai Puhui Hospital of Integrated Traditional Chinese and Western Medicine, Yantai 264004, Shandong Province, China; ^6^Guang'anmen Hospital China Academy of Chinese Medical Science, Beijing 100053, China

## Abstract

Myocardial infarction can lead to ventricular remodeling and arrhythmia, which is closely related to nerve remodeling. Our previous study found that *Yiqi Huoxue* decoction (YQHX) can improve ventricular remodeling and reduce myocardial damage. Therefore, in this study, we observed the effect of YQHX on cardiac neural remodeling and cardiomyocyte hypertrophy and its possible mechanism. This research is composed of two parts: animal and H9c2 cells experiments. The animal model of acute myocardial infarction was established by ligating the left anterior descending coronary artery in Sprague Dawley (SD) rats. H9c2 cells were placed in 94% N_2_, 5% CO_2_, and 1% O_2_ hypoxic environment for 12 hours to replicate the hypoglycemic hypoxia model. The experimental results showed that, compared with the MI group, YQHX can significantly improve heart function after myocardial infarction and reduce nerve remodeling and myocardial hypertrophy. Pathological structure observation demonstrated reducing myocardial tissue damage and decreasing of cell cross-sectional area, diameter, and circumference. The positive rate of TH declined apparently, and the sympathetic nerve density was lower than that of the MI group. After YQHX was given for 28 days, the proneural remodeling factors TH, NGF, and GAP43 in the marginal zone of infarction and stellate ganglion decreased obviously while the inhibitory nerve remodeling factor Sema-3A increased. The myocardial hypertrophic protein ANP and *β*-MHC were also significantly inhibited with p-ERK1/2 protein expression level prominently reduced. There was no difference between the YQHX group and the Meto group. After myocardial infarction, nerve remodeling was seen in the marginal area of infarction and stellate ganglion, and the neuropeptides released by which promoted myocardial hypertrophy. The mechanism may be related to the ERK1/2 signaling pathway. YQHX could regulate the ERK1/2 signaling pathway, inhibit the release of nerve remodeling factors and myocardial hypertrophy protein to reduce nerve remodeling, and relieve myocardial hypertrophy.

## 1. Introduction

In cardiovascular diseases, especially ischemic heart disease, abnormal autonomic nerve regulation plays a vital role in the occurrence, development, and prognosis of the disease [1, 2]. Myocardial infarction (MI) can lead to ventricular remodeling, arrhythmia, and sudden death. It is an important cause of death, which is closely related to nerve remodeling [3, 4]. Intramyocardial nerve sprouts in the marginal area of the infarct and remote normal tissue have been observed in both animals and humans [5, 6]. Recent research identified that the stress state can bring about the activation of sympathetic nervous system with the enhancement of excitability. This activation may be one of the rapid compensation mechanisms at the early stage after MI, but the long-term activation at a high level of the sympathetic nervous system is an important facilitator in the occurrence of ventricular remodeling and deterioration of the disease due to its adverse effect on the heart itself and cardiac function [7]. After the occurrence of acute myocardial infarction (AMI), the nerves that innervate the heart are damaged. Passing the acute phase, with the action of nerve factors, such as nerve growth factor (NGF) and growth related protein 43 (GAP43), the damaged nerves regenerate and gradually repair. During the repair process, a variety of local and circulating nerve factors including NGF are involved, manifesting as excessive regeneration of nerve sheath cells and axons, especially in the peripheral area of the infarction, which is a sensitive zone for tissue repair and regeneration after MI [8, 9]. Neural remodeling factors play a very important role in this process, reflecting the activity of sympathetic nerves [10], promoting the repair of sympathetic nerve damage [11], and avoiding excessive growth [12]. The hyperproliferation of sympathetic nerve endings will release a large amount of catecholamines and neuropeptides (NE, NPY), which can promote cardiac hypertrophy and ventricular hypertrophy, ultimately leading to heart failure and sudden death.

The stellate ganglion (SG) is formed by the fusion of the lower neck, the first or the second thoracic sympathetic ganglion, belonging to the sympathetic ganglion functionally [13]. The left stellate ganglion (LSG) mainly innervates the left side of the heart and most of the left and right ventricles. The increasing of the excitability of this side ganglion will enhance the susceptibility to arrhythmia. After MI, with the hypoxia, injury, and lytic necrosis of cardiomyocytes, excessive proliferation of the left stellate ganglion would occur, which was called stellate ganglion remodeling by some scholars [14]. NGF rapidly released and the concentration of NGF and GAP43 in the area around the infarction increased immediately after myocardial infarction. Both of them were transferred to the left stellate ganglion, leading to nerve growth in the left ventricular and promoting sympathetic nerve remodeling. This was also verified in Nori's experimental observation [15]. Therefore, in this study, we also observed the changes of neurofactors in stellate ganglion.

*Yiqi Huoxue* decoction (YQHX) has been widely used for the treatment of myocardial diseases considering its efficacy and safety [16]. Previous studies have shown that *Yiqi Huoxue* decoction can improve ventricular remodeling [17] and mitochondrial function [18], regulate energy metabolism [19, 20], and relieve myocardial infarction damage in rats. Although YQHX can regulate arrhythmia and improve cardiac function in clinical treatment, experimental studies have also shown that YQHX can improve ventricular remodeling, but whether its mechanism of action is related to neural remodeling is still unclear. In summary, we observed action chain of post-myocardial infarction neural remodeling—overactivation of the sympathetic nervous system—large release of neuropeptide Y—promoting cardiomyocyte hypertrophy. The changes in factors related to nerve remodeling and myocardial hypertrophy after myocardial infarction and the role of YQHX were observed as well.

## 2. Materials and Methods

### 2.1. Ethical Approval

According to the “Guidelines for the Care and Use of Laboratory Animals” published by the National Institutes of Health (NIH Publication no. 85-23, revised in 1996), the experiment was reviewed and approved by the Medical and Laboratory Animal Ethics Committee of Beijing University of Chinese Medicine (ethics number: BUCM-4-2018071701-3002).

### 2.2. Herb Preparation

YQHX is composed of five herbs: *Astragalus membranaceus*, *Angelica sinensis*, *Ligusticum chuanxiong Hort*, *Panax notoginseng*, and *Panax ginseng C. A. Meyer*. All the herbs were purchased from Dongzhimen Hospital of Beijing University of Chinese Medicine. The team used HPLC-LTQ Orbitrap MS technique with DDA-MS2 data acquisition method and literature to systematically study the components of the aqueous extracts of YQHX and inferred a total of 87 compounds, the structural types of which are mainly triterpenoid saponins and flavonoids, including ginsenosides, panaxosides, floralginsenoside, quinquenoside, and astragaloside [18].

The components of YQHX were extracted by refluxing with boiling distilled water (1 : 10, g/mL) three times. After filtration, the water extract is concentrated to a constant volume. The concentrated extract is used in animal experiments and prepared into powder by freeze-drying in vacuum for cell experiments.

According to the recommended raw drug dose of 96 g/day for humans, the dose for rats is 6 times higher than the human equivalent dose ratio, so the selected drug dose is 8.2 g/kg, which has been shown to be effective in our previous studies. The cell concentration of YQHX was obtained by cck-8 assay and cell damage, screened from a gradient of varying concentrations from 50 *μ*g/ml to 800 *μ*g/ml [18].

### 2.3. Animal Model Establishment and Group Administration

Adult male SPF-grade Sprague Dawley (SD) rats (200 ± 20 g) were purchased from Beijing Weitong Lihua Laboratory Animal Technology Co., Ltd. (license number: SCXK2016-0006). The animals were adaptively fed at the Animal Experiment Center of Beijing University of Chinese Medicine for 3 days before myocardial infarction surgery. During the feeding period, the rats ate freely with regular diet and distilled water. The temperature and humidity in the laboratory were constant. The rat model of myocardial infarction was prepared as described previously [21]. The experimental surgical procedure was sterile, and the rats were anesthetized with 1% sodium pentobarbital (40 mg/kg) intraperitoneally. After tracheal intubation, a small animal ventilator was connected with a respiratory rate of 85 times/min and a respiratory ratio of 1 : 2. The surgical area is prepared and disinfected. The muscles were bluntly separated between the third and fourth ribs to expose the chest cavity and heart. When preparing the model, we ligated the left anterior descending coronary artery with 5-0 nylon sutures and then closed the thoracic cavity layer by layer. Except for LAD ligation, the same procedures were conducted to the rats used for the control. The successful modeling of myocardial infarction was verified by ST segment significantly elevating under the monitoring of electrocardiogram (ECG) limb leads (Shanghai Medical Electronic Instrument Factory).

The rats were randomly divided into sham-operated group (10 rats) and infarct group. Rats in the infarct group were tested by ECG on the second day after surgery, and more than 4 Q-wave numbers meet the experimental requirements. The rats with the same Q-wave number were randomly assigned to each group. Ten rats that were only threaded without ligation were used as sham operation group (Sham). SD rats that were successfully ligated were randomly divided into three groups: myocardial infarction model (MI, *N* = 10), myocardial infarction model + YQHX (YQHX, *N* = 10), and myocardial infarction model + metoprolol (Meto, *N* = 10). Gavage was started on the second day after MI surgery, the dose of YQHX was 8.2 g/kg/d, and the dose of metoprolol (AstraZeneca Pharmaceutical Co., Ltd., National Medicine Standard H32025391, production batch number: 1805A23) was 5 mg/kg/d. Sham and MI groups were given an equal volume of distilled water. All indicators were observed after 28 days of intragastric administration.

### 2.4. Cell Culture and Group Administration

H9c2 cells were cultured in the medium containing 10% fetal bovine serum and 1% penicillin and placed in a 5% CO_2_ incubator at 37°C. According to the previous research results, the most appropriate concentration was selected to prepare a 200 *μ*g/mL drug working solution [22]. Due to its best antioxidant effect, the moderate and mild damage of myocardial cells caused by 12 h hypoxia was effectively resisted. When the cells reached 80% of growth, they were divided into (1) control group (c): cells were cultured in normoxic condition; (2) model group (m): cells were placed in 94% N_2_, 5% CO_2_, and 1% O_2_ hypoxic environment for 12 h; (3) model + 200 *μ*g/mL YQHX group (YQHX): YQHX freeze-dried powder was dissolved in DMEM at the concentration of 200 *μ*g/mL while the cells were placed in hypoxic environment; (4) model + ERK1/2 inhibitor PD98059 group (PD): pretreated cells with ERK inhibitor PD98059 (10 *μ*M) for 1 h [23]; (5) model + 200 *μ*g/mL YQHX + ERK1/2 inhibitor PD98059 group (YQHX + PD): pretreated cells with ERK inhibitor PD98059 (10 *μ*M) for 1 h; YQHX was dissolved in DMEM at the concentration of 200 *μ*g/mL, and cells were exposed to hypoxia.

### 2.5. Echocardiogram

The rats were examined by echocardiography (Visual Sonics, Vevo770, Canada) before sampling. The rats were anesthetized with intraperitoneal injection of 1% sodium pentobarbital, and the chest skin was prepared in the supine position. The normal physiological state was maintained during the ultrasound process. The average of 3 consecutive cardiac cycles was taken from each mouse. We detected left ventricular ejection fraction (EF), left ventricular short axis rate (FS), left ventricular end diastolic diameter (LVIDd), and left ventricular end systolic diameter (LVIDs).

### 2.6. Hematoxylin and Eosin (HE) Staining

After the myocardial tissue was fixed, dehydrated, embedded, and sectioned (4 *μ*m thickness), conventional HE staining was performed. The sections were deparaffinized and dehydrated in descending gradient ethanol. The nuclei were stained with Harris hematoxylin solution and differentiated with 1% hydrochloric acid ethanol. Staining was continued with 1% red ethanol solution followed by gradient ethanol dehydration. Finally, the sections were observed under microscope and photographed for analysis.

### 2.7. Immunohistochemical

The paraffin sections were deparaffinized and placed in a repair box filled with EDTA antigen retrieval buffer (pH 8.0) in a steamer for antigen retrieval. The slides were placed in PBS (pH 7.4) and washed 3 times by shook on a decolorizing shaker for 5 minutes each time. We blocked endogenous peroxidase, drew a circle, and sealed them with serum. Primary antibody was added: the blocking solution was gently shook off, a certain ratio of primary antibody was added to the slice, and the slice was placed flat in a humid box at 4°C incubating overnight. The secondary antibody was added: the slide was placed in PBS (pH 7.4) on a decolorizing shaker and washed 3 times, 5 min per time. After the sections were slightly dried, the secondary antibody was added corresponding to the primary antibody in the circle to cover the tissue. Then, the sections were incubated at room temperature for 1 h. Next, the tissue was washed and HRP was incubated. To continue, it was washed for DAB color development: the PBS solution was removed, 50–200 *μ*L of fresh DAB solution was added to each slice, and the microscope was used to control color development. Hematoxylin counterstained the nucleus.

### 2.8. Determination of LDH, MDA, and SOD Levels in Cell Supernatant

H9c2 cardiomyocytes were seeded in a 6-well plate. After 24 hours of cell adhesion, the cell culture medium of each group was collected after drug intervention and hypoxia. The supernatant was centrifuged at 3000 r/min for 20 min and taken for further operation according to the instructions of the LDH, MDA, and SOD kits. The OD value and optical density value were measured by using a spectrophotometer.

### 2.9. Hoechst 33258 Test

After 12 hours of hypoxia, suction was given to the culture medium, and the cells were rinsed in PBS at 37°C for 3 times and fixed in 4% paraformaldehyde for 15–20 minutes. Another round of rinse in PBS was conducted for 3 times, and then cells were covered with a small amount of Hoechst 33258 working solution. The cells were placed at room temperature for 3–5 minutes. After washing the staining solution, we observed the cells under fluorescence microscope. The nuclei of apoptotic cells showed dense staining or fragmentary dense staining.

### 2.10. Western Blotting

1 mL RIPA lysis buffer solution was added to H9c2 cell and tissue samples for homogenization. Samples were lysed on ice for 30 minutes for protein extraction, and BCA protein assay kit was utilized to quantify the obtained protein. The total amount of loaded protein was 40 ug, 20 ul. The protein was separated on a gel prepared with an appropriate percentage to detect the molecular weight of the target protein and then followed by electrophoresis and electrotransformation. The PVDF membrane after electroporation was immersed in a TBS-T blocking solution containing 5% milk for sealing. Then, it was incubated overnight combined with NGF antibody (ab52918, Abcam), GAP43 antibody (ab75810, Abcam), Semaphorin 3A antibody (ab11370, Abcam), Tyrosine Hydroxylase antibody (ab112, Abcam), ERK antibody (9102, CST), p-ERK antibody (9101, CST), c-fos antibody (ab7963, Abcam), c-Myc antibody (ab39688, Abcam), and actin antibody (ab8226, Abcam). The relative expression level of protein was detected by electrochemiluminescence and gray-scale scanning gel imaging analysis.

### 2.11. Statistical Analysis

The data obtained in the experiment was processed with SPASS22.0 software. One-way analysis of variance (one-way ANOVA) was used, and *P* < 0.05 was used as the statistically significant standard.

## 3. Results

### 3.1. Changes of Cardiac Structure and Function in Rats with Myocardial Infarction

**Figure 1:**
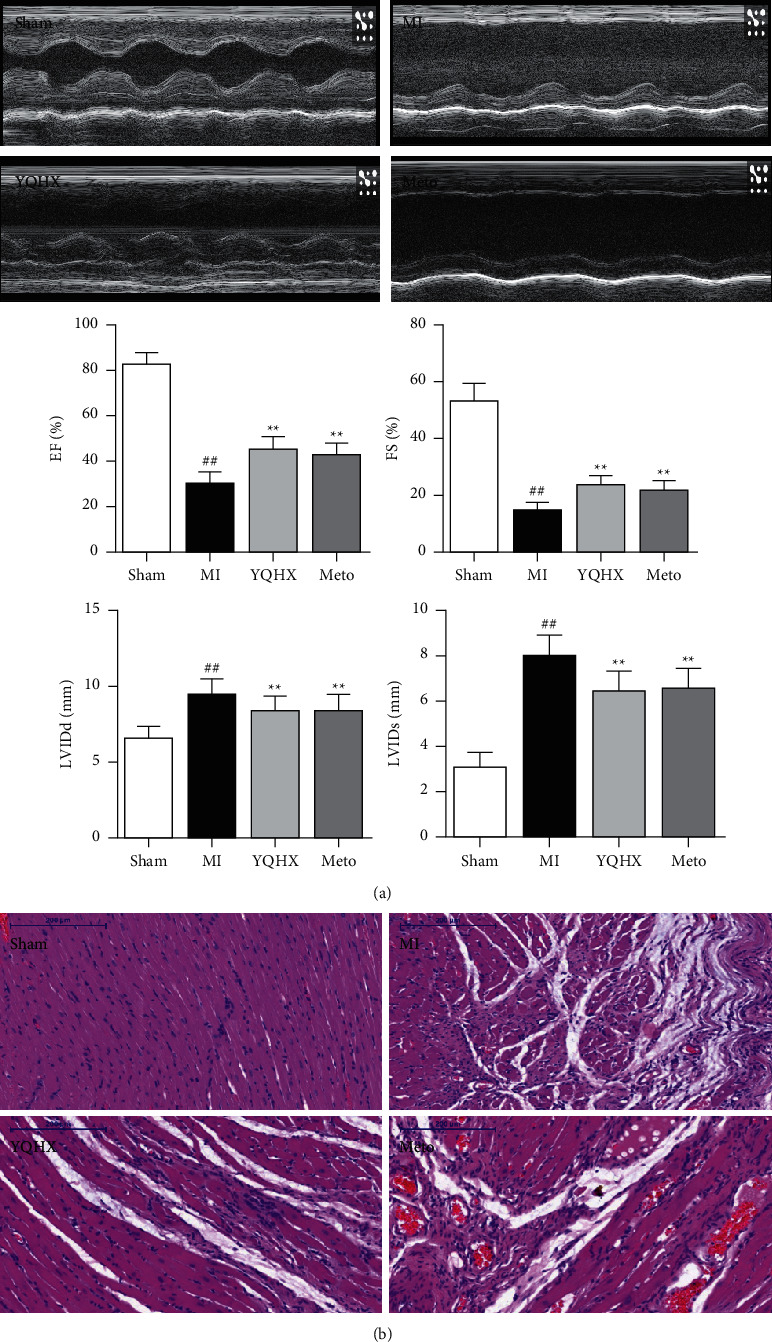
(a) Echocardiogram and cardiac function of rats in each group 28 days later (*N* = 9). ^##^*P* < 0.01, vs. the sham operation group; ^*∗∗*^*P* < 0.01, vs. MI model group. (b) Histopathological hematoxylin and eosin staining of infarct marginal area tissue of rats with myocardial infarction in each group (scale bar = 200 *μ*m).

Echocardiography is an important and direct method to evaluate the function of the heart. It can be seen from Figure 1(a) that the EF and FS of the rats in the MI group significantly decreased after acute myocardial infarction. The LVIDs and LVIDd significantly increased, and the cardiac function was severely damaged. After 28-day intervention of YQHX and Meto, the values of EF and FS increased significantly while LVIDs and LVIDd decreased. The difference was obvious. However, there is no significant difference between YQHX and metoprolol in efficacy. But the ejection fraction and left ventricular systolic inner diameter thickness in YQHX group were slightly better than those in metoprolol group, indicating that the efficacy of YQHX is slightly better than metoprolol in the chronic phase. HE staining is one of the methods used to observe the morphological and structural changes of myocardial tissue. Compared with the Sham group, the myocardial cells of MI group arranged disorderly and the quantity decreased. The cell morphology was abnormal with scattering nuclei, a large number of inflammatory cells infiltrating with enlarged intercellular space. In the YQHX and Meto intervention groups, the morphological structure of the infarct marginal zone was significantly superior to that of the MI group, and more viable cardiomyocytes were seen (Figure 1(b)). In summary, YQHX can significantly improve the cardiac function of rats after myocardial infarction and relieve myocardial damage in the marginal zone of infarction.

### 3.2. Effect of YQHX on Nerve Remodeling Factors in Marginal Area of Myocardial Infarction Rats

**Figure 2:**
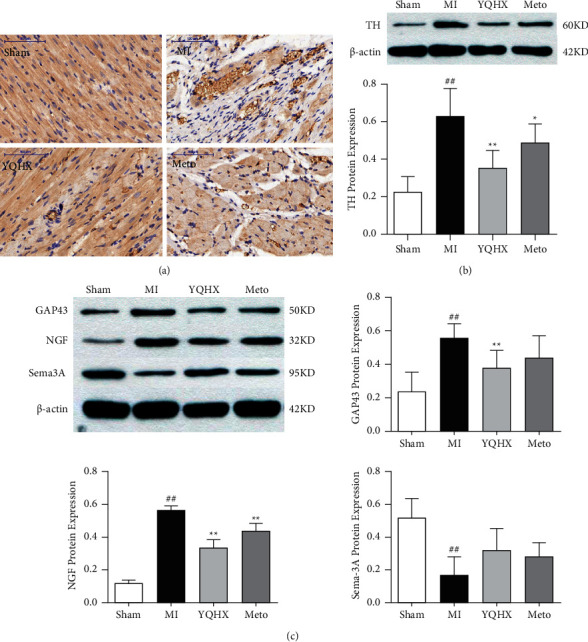
(a) Immunohistochemical staining to observe the TH staining positive sympathetic fibers in the infarct marginal zone of myocardial infarction rats (scale bar = 100 *μ*m). (b) The expression of TH in the infarct marginal area tissue of each group. ^##^*P* < 0.01, vs. the sham operation group; ^*∗∗*^*P* < 0.01, vs. MI model group. (c) The effect of YQHX on the expression of GAP43, NGF, and Sema-3A. ^##^*P* < 0.01, vs. the sham operation group; ^*∗∗*^*P* < 0.01, vs. MI model group.

Nori and his colleagues [15] have found that tyrosine hydroxylase (TH) was the majority of regenerated nerve fibers after myocardial infarction, indicating that the regenerated nerves are dominated by mature sympathetic nerves. Our experiments also verified this research result (Figure 2(a)). Positive nerve fibers in the Sham group were rare or absent 28 days after myocardial infarction, evenly distributing among the myocardial fibers, consistent with the direction of myocardial cells; the TH-positive nerve fibers in the MI group significantly increased in density with thick shape and disordered distribution. After YQHX and Meto treatment, the density of TH-positive nerve fibers significantly decreased, and their morphology and distribution were more normal than those in the MI group. The content of TH protein in myocardial tissue was lower (Figure 2(b)). NGF and GAP43 are nerve growth factors, which can cause excessive regeneration and uneven density of cardiac nerves after myocardial infarction while Sema-3A is a nerve growth inhibitor, which can inhibit the excessive growth of sympathetic nerves. The protein content of NGF and GAP43 in the marginal area of the MI group increased significantly while the protein level of Sema-3A decreased 28 days after myocardial infarction (Figure 2(c)). After YQHX and Meto intervention, the expression of NGF and GAP43 was reduced to promote their return to the normal range. Although the Sema-3A protein level increased in the medication group, it did not effectively inhibit nerve regeneration and remodeling. To sum up, YQHX can significantly moderate the neurological factors to the normal range, and the efficacy of YQHX is better than metoprolol in the chronic phase.

### 3.3. Effect of YQHX on Nerve Factors in Stellate Ganglion of Myocardial Infarction Rats

**Figure 3:**
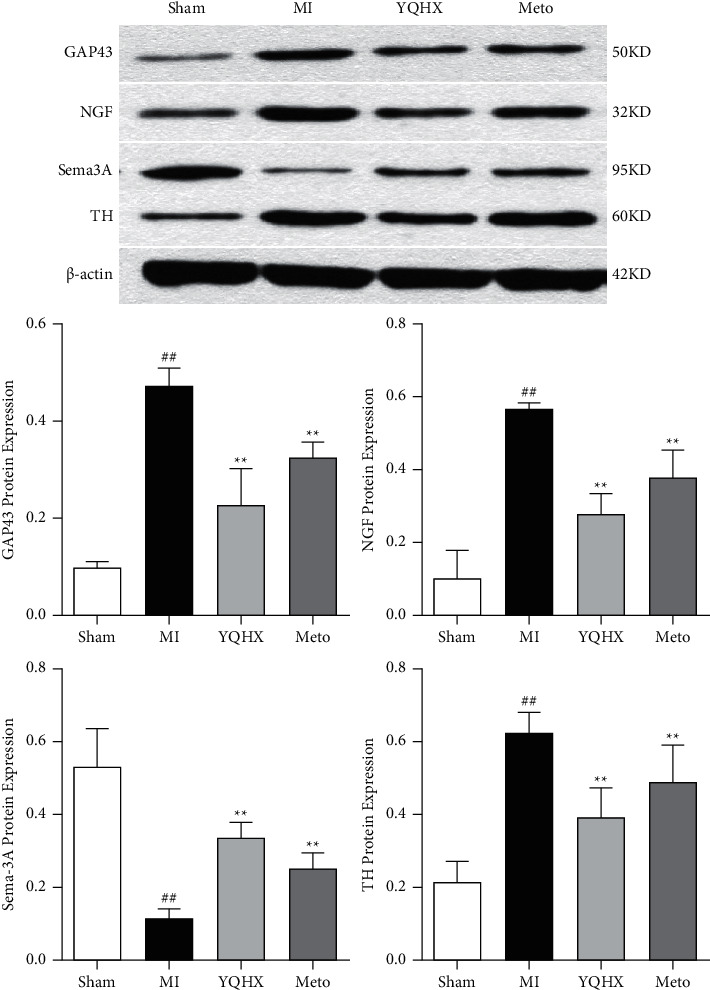
The expression of nerve factors in stellate ganglia. ^##^*P* < 0.01, vs. the sham operation group; ^*∗∗*^*P* < 0.01, vs. MI model group.

In animal models of ischemia-reperfusion injury, nerve remodeling is related to the expression and release of neurotrophic factors (including NGF), which can be transported to stellate ganglia through nerve bundles [24]. Zhou et al. [25] found that the level of nerve growth factor and growth related protein 43 (GAP43) messenger ribonucleic acid in the left stellate ganglion increased significantly after myocardial infarction, thereby increasing the density of cardiac nerves. These findings indicate that the stellate ganglion is closely related to cardiac nerve function. The cardiac nerve remodeling may be regulated by neurofactors of the stellate ganglion. Therefore, in this experiment, the expression of nerve factors in the stellate ganglion after myocardial infarction was observed. Interestingly, the results showed that the variation trend of nerve factors is consistent with that in the heart tissue (Figure 3).

### 3.4. Effect of YQHX on NPY in Rats after Myocardial Infarction

**Figure 4:**
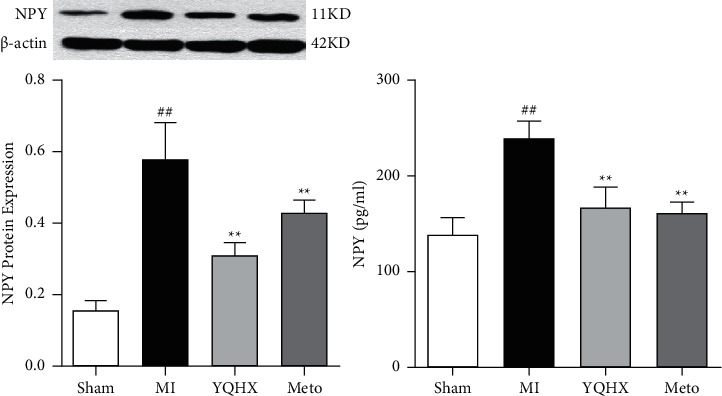
The expression of NPY in the infarct marginal zone tissue and serum of each group. ^##^*P* < 0.01, vs. the sham operation group; ^*∗∗*^*P* < 0.01, vs. MI model group.

NPY is a polypeptide widely present in the central and peripheral nervous system [26]. It can promote cardiomyocyte hypertrophy and participate in cardiac hypertrophy and heart and blood vessels remodeling [27]. After myocardial infarction, the sympathetic nervous system is activated with the sympathetic nerve fibers releasing a large amount of NPY, which can aggravate the excessive excitement and uneven distribution of the myocardial sympathetic nerve. When myocardial ischemia and hypoxia occur, the sympathetic nerve becomes excited to increase the release of neurotransmitter, accompanied by a rising in synthesis. NPY can strongly contract the coronary arteries, thereby aggravating sympathetic nerve remodeling and causing or aggravating coronary artery disease. In the peripheral nervous system, NPY, as a neurotransmitter or modulator, exerts its physiological regulation effect, regulating the body's cardiovascular activities from multifaced aspects. Therefore, the content of NPY in myocardial tissue and serum was detected in this experiment. The results showed that the content of NPY in the tissue and plasma of the marginal area of infarction increased significantly after myocardial infarction (Figure 4). After 28 days of intervention with YQHX and Meto, the level of NPY in tissue and serum decreased, which was beneficial to reduce the excessive innervation of the nervous system to the myocardial tissue and alleviate the progress of myocardial hypertrophy.

### 3.5. Effect of YQHX on Cardiomyocyte Hypertrophy in Myocardial Infarction Rats

**Figure 5:**
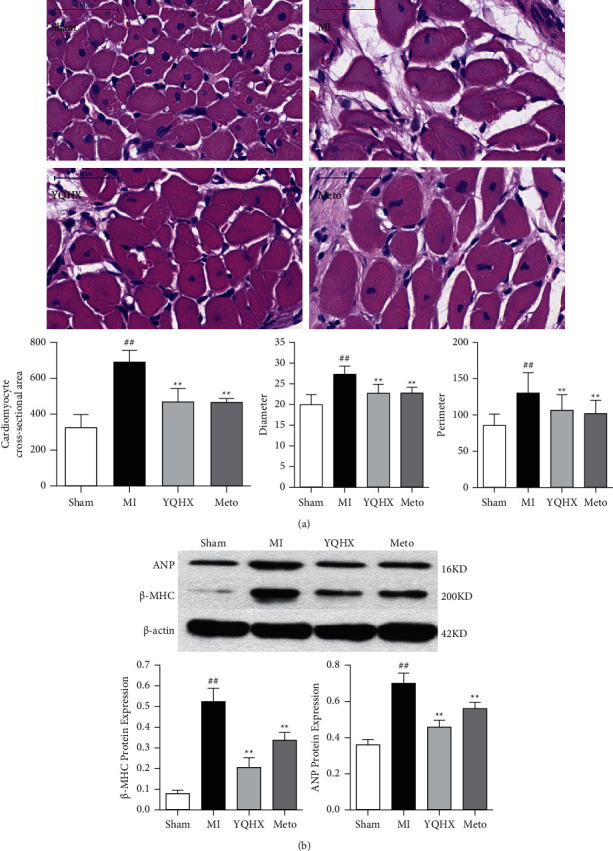
(a) Observation of the cross section of cardiomyocytes stained with histopathological hematoxylin and eosin and the effect of YQHX on the cross-sectional area, average diameter, and perimeter of cardiomyocytes (scale bar = 50 *μ*m). (b) The effect of YQHX on the expression of ANP and *β*-MHC protein in the marginal zone of myocardial infarction in rats. ^##^*P* < 0.01, vs. the sham operation group; ^*∗∗*^*P* < 0.01, vs. MI model group.

Myocardial hypertrophy is a basic response of cardiomyocytes to common clinical diseases such as acute myocardial infarction, hypertension, and congenital heart disease. *β*-MHC gene is one of the marker genes of cardiac hypertrophy. The protein expression of *β*-MHC reflects the changes of ventricular hypertrophy to a certain extent, and it is often used together with ANP as an index to evaluate myocardial hypertrophy. In this study, Image-Pro Plus software was used to measure the cross-sectional area, diameter, and perimeter of cardiomyocytes. After intragastric administration for 28 days, the myocardial intercellular space became larger, the cells swelled, the nucleus dissociated, the cell cross-sectional area became larger, and the fibrous and collagen tissue proliferated in MI group. The cell swelling in the YQHX group and Meto intervention group could be alleviated (Figure 5(a)). *β*-MHC and ANP protein level can be significantly reduced after medical intervention (Figure 5(b)), indicating that YQHX can relieve myocardial hypertrophy after myocardial infarction.

### 3.6. Effect of YQHX on ERK1/2 and p-ERK1/2 in Myocardial Infarction Rats

**Figure 6:**
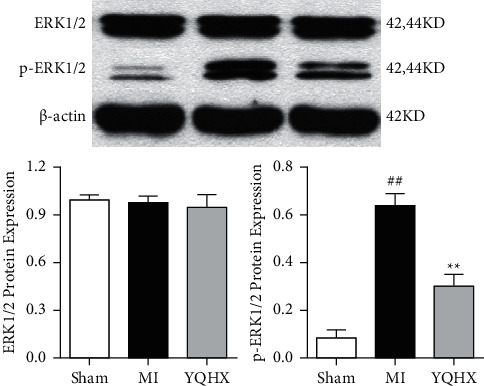
The effect of YQHX on ERK1/2 protein and its phosphorylation. ^##^*P* < 0.01, vs. the sham operation group; ^*∗∗*^*P* < 0.01, vs. MI model group.

ERK1/2 signaling pathway is one of the important signaling pathways for ventricular remodeling after myocardial infarction, which plays an important role in regulating cardiomyocytes and neurons. Based on the regulatory effect of YQHX on post-myocardial infarction neural related factors and myocardial hypertrophy, we observed the expression of ERK1/2 and p-ERK1/2 proteins in the infarct marginal zone of rats after myocardial infarction and the effect of YQHX. The results demonstrated that the expression of p-ERK1/2 protein increased significantly after MI, and YQHX could reduce the expression (Figure 6). Therefore, it is speculated that YQHX may regulate the expression of nerve related factors through the ERK1/2 phosphorylation signal pathway, reduce the excessive activation of sympathetic nerves, alleviate the progress of cardiac hypertrophy, and play a role in protecting the heart.

### 3.7. The Protective Effect of YQHX on Hypoxic H9c2 Cells and the Regulation of NGF

**Figure 7:**
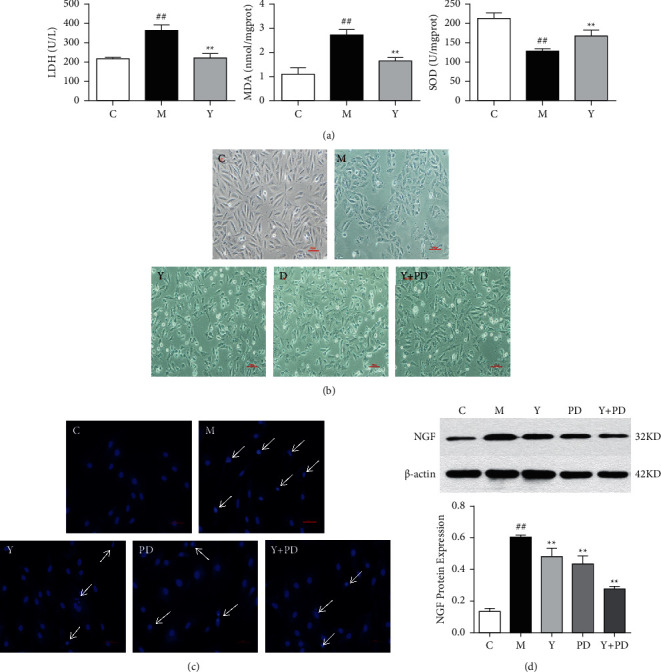
(a) The effect of YQHX on the levels of SOD, MDA, and LDH in cell supernatant. ^##^*P* < 0.01, vs. the C group; ^*∗∗*^*P* < 0.01, vs. the M group. (b) Morphological changes of cardiomyocytes in each group (scale bar = 100 *μ*m). (c) Hoechst 33258 staining showed the apoptosis of each group (scale bar = 100 *μ*m). (d) NGF protein expression in each group of cells. ^##^*P* < 0.01, vs. the C group; ^*∗∗*^*P* < 0.01, vs. the M group.

In order to evaluate the hypoxic injury, we observed the effect of YQHX on LDH, MDA, and SOD of hypoxic cardiomyocytes. Superoxide dismutase (SOD) is closely related to cell oxidative metabolism and is the first line of defense to prevent damage from oxidative stress. It can remove excess oxygen free radicals to keep the body in a balanced state. Its activity can reflect the body's ability to eliminate oxygen free radicals and indirectly reflect the body's protective effect on oxidative damaged cells. Malondialdehyde (MDA) is a product of lipid peroxidation between oxidative stress and unsaturated fatty acids in cell membranes and is then decomposed by oxidase. The expression of MDA is similar to SOD, which reflects the degree of lipid peroxidation. The excessive content indicates that it is seriously attacked by oxygen free radicals, leading to cell damage [28]. Lactate dehydrogenase (LDH) is a marker enzyme in cardiomyocytes. Cell damage leads to the release of large amounts of LDH. Therefore, the increase of LDH content in cell supernatant can reflect the degree of myocardial cell injury. The results showed that after 12 h of hypoxia, YQHX could reduce the level of LDH and MDA and increase the content of SOD (Figure 7(a)), which indicated that YQHX could alleviate oxidative stress injury and protect the integrity of cell membrane.

We used ERK1/2 inhibitor PD98059 to inhibit the expression of this protein pathway and observed the effect on hypoxic cells. Under the microscope, it can be seen that the cells in group C were fusiform, with clear boundaries, complete morphology, and uniform distribution; the number of cells in group M decreased with polygonal morphology and nuclear shrinkage; the damage of cells in group Y was less than that in group M (Figure 7(b)). Hoechst staining showed that the cells in group C were evenly stained with normal shape and size; in group M, the cells were densely stained with fragmentation; in YQHX and inhibitor intervention groups, the cell fragments were reduced and the morphology tended to be normal (Figure 7(c)). Subsequently, the changes of nerve growth factor in H9c2 cells were observed. YQHX can reduce the expression of NGF protein in cardiomyocytes after hypoxia (Figure 7(d)), and this effect may be related to the inhibition of ERK1/2 signaling pathway.

### 3.8. Effect of YQHX on ERK1/2 Signal Pathway in Hypoxic H9c2 Cells

**Figure 8:**
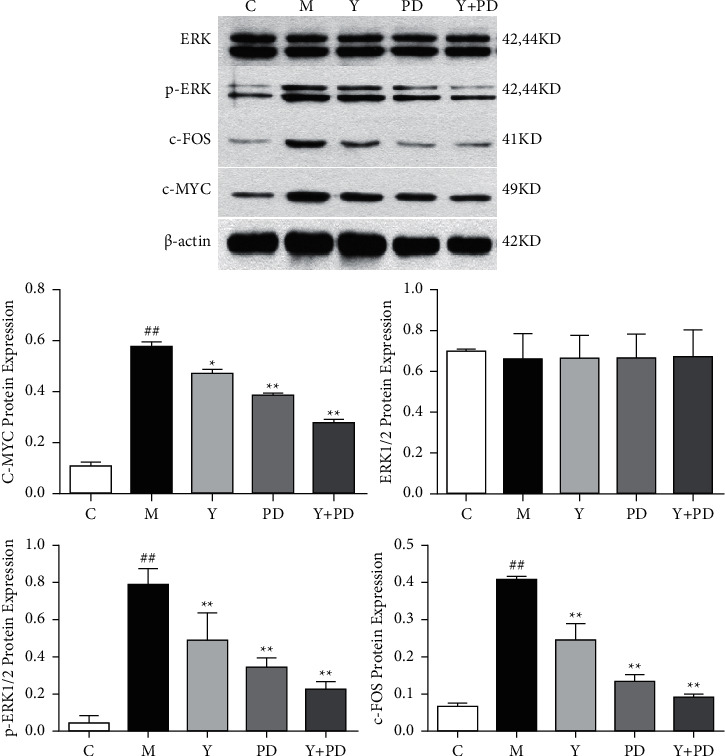
The expression of ERK1/2 signaling pathway related proteins in each group of cells. ^##^*P* < 0.01, vs. the C group; ^*∗*^*P* < 0.05, vs. the M group; ^*∗∗*^*P* < 0.01, vs. the M group.

In the previous animal experiments, we found that YQHX can inhibit the occurrence of neural remodeling and the activation of the sympathetic nervous system, thereby inhibiting cardiomyocyte hypertrophy and protecting cardiomyocytes. Its protective effect may be related to the ERK1/2 signaling pathway. In order to further study the mechanism of YQHX, we used ERK1/2 inhibitor PD98059 to inhibit the expression of ERK1/2 and its phosphorylation. ERK1/2 signaling pathway related proteins p-ERK1/2, c-fos, and c-myc significantly decreased after YQHX and inhibitor intervention (Figure 8). The above results indicated that YQHX can regulate the expression of nerve growth factor by regulating the ERK1/2 signaling pathway, reduce excessive nerve regeneration and remodeling, and slow down the development of myocardial hypertrophy.

## 4. Discussion

Nerve remodeling can occur at the early stage of post-myocardial infarction. With the occurrence of ventricular remodeling, it will cause damage, necrosis, and remodeling of sympathetic nerves in the same area. Studies [29–33] have found that sympathetic nerve remodeling after MI is an important factor and mechanism that triggers, initiates, and even maintains malignant arrhythmia and that the changes of cardiac nerves precede the abnormalities of cardiac structure and function. Whether the features of multicomponent, multitarget, and multipathway of traditional Chinese medicine are related to the regulation of nerve internal environment needs further study. YQHX is composed of five herbs of *Astragalus membranaceus*, *Angelica sinensis*, *Ligusticum chuanxiong Hort*, *Panax notoginseng*, and *Panax ginseng C. A. Meyer*. It is widely used in the treatment of ischemic cardiomyopathy and can significantly improve the clinical symptoms and signs of patients. It has achieved good curative effects in experimental animal studies and clinical applications [34]. YQHX is an empirical prescription for clinical treatment of myocardial ischemia based on the theory of qi and blood in Chinese medicine. It has the effects of invigorating qi, nourishing heart, and activating blood circulation. In this experiment, the classic left anterior descending coronary artery ligation was used to establish a rat model of myocardial infarction to study the protective effect of YQHX on myocardial ischemia and hypoxia. As a *β*-blocker, metoprolol has sufficient evidence-based medical evidence to verify that it can block or delay myocardial remodeling and improve long-term prognosis [35, 36]. The effects of YQHX and metoprolol on cardiac function after myocardial infarction were observed. The results showed that taking YQHX for 28 days can significantly improve the EF, FS, LVIDs, and LVIDd of rats after myocardial infarction and improve the pathological structure of the infarct marginal area tissue. There is no significant difference between YQHX and metoprolol, but its efficacy is superior to metoprolol in the chronic phase.

After acute myocardial infarction, the sympathetic nerve that dominates the heart regenerates after being injured. The regenerated nerves are different in structure and function from normal parts. It is heterogeneous excessive regeneration and nerve remodeling, often accompanied by ventricular remodeling and electrical remodeling, which can promote the occurrence of malignant arrhythmia and sudden death. In a series of studies in animals and humans, it was found that neurons in the stellate ganglion expanded, nerve sprouting, and nerve signals increased after MI [37–39]. The changes of nerve factors at the stellate ganglion are closely related to cardiac nerve remodeling after MI [40]. Han et al. [37] found that the stellate ganglion nerve activity (SGNA) increased rapidly after MI. However, the increase in SGNA was related to the sprouting of nerves in the myocardium and the increase in the size and synaptic density of the stellate ganglion neurons. Anatomy revealed that the sympathetic nerve fibers from the stellate ganglia on the left and right sides were mixed with the parasympathetic nerve fibers from the vagus nerve [41]. After myocardial infarction, neurotrophic factors can be transported to the stellate ganglion through nerve bundles [24, 25]. When stellate ganglion block is used, it can reduce myocardial damage [42]. All these indicate that the stellate ganglion can affect the cardiac nerves through the release of mixed nerves and/or nerve factors. In short, after myocardial damage, stellate ganglion nerve remodeling and increased sympathetic nerve activity may be a key factor in cardiac sympathetic nerve dysfunction [38] and cardiac nerve remodeling. The distribution and density of TH-positive sympathetic fibers can directly show the state of nerve remodeling. Tyrosine hydroxylase (TH) distributes in the cytoplasm of adrenergic axons and is the rate limiting enzyme of norepinephrine synthesis with high specificity [43]. TH-positive expression can represent the distribution of sympathetic nerves in the heart, as an important marker for evaluating cardiac sympathetic nerve regeneration, not only as a marker for sympathetic nerve endings, but also as an indirect indicator of sympathetic nerve activity [44]. In this experiment, by observing the protein content, morphology, and distribution density of TH, we found that, after myocardial infarction, the sympathetic nerves were overactive and proliferated [29]. NGF can assist cardiomyocytes to get close to sympathetic nerve neurons to generate new neurons of sympathetic nerve fibers and then metabolize new nerve fibers [45]. Studies have found that NGF can also promote the occurrence and development of neuritis and induce excessive cardiac sympathetic nerve activity [46], indicating that NGF plays an important role in physiological and pathological conditions. Some research [25] identified that AMI can cause an immediate increase in local NGF concentration within 3.5 hours and then increase the expression of growth associated protein (GAP43) in the infarct area. GAP43 is a neuron specific protein, which is synthesized by neuron cell bodies and widely exists in the axons of neurons [47]. In mature or stable neurons, GAP43 usually exists in the form of no expression or low expression, but its expression increases throughout the development and regeneration of the nervous system, so the expression of GAP43 also symbolizes the growth of nerves [48]. A number of experiments [49, 50] have verified that the expression of GAP43 factor and NGF increase at the same time, indicating the rapid growth of related nerves, especially after myocardial infarction. Zhou et al. [25] found that the level of NGF and GAP43 messenger ribonucleic acid in the LSG increased significantly after MI, thereby increasing the density of cardiac nerves. Studies have found that sympathetic nerve remodeling after MI has been observed in patients with myocardial infarction and animal models [51, 52], and the densities of TH and GAP43 positive nerve fibers have increased. Subsequent studies [53, 54] have shown that new sprouting nerves are more obvious in the area around the infarction. Therefore, the upregulation of the two proteins is considered to represent sympathetic remodeling [55]. Sema-3A is a neurosuppressive factor. It is the only factor that helps to improve sympathetic nerve remodeling by inhibiting sympathetic nerve regeneration to prevent overinnervation. Sema-3A plays an important role in maintaining the balance of cardiac autonomic nerve during the process of remodeling after myocardial infarction [56]. In this study, YQHX can significantly regulate nerve remodeling related factors and reduce the protein content of TH, NGF, and GAP43 in the marginal area of infarction and the stellate ganglion in rats with myocardial infarction. In experimental studies in dogs with myocardial infarction, increased densities of TH and GAP43 expression were also found, and their expression was reduced by technical interventions to improve left ventricular structure and sympathetic remodeling after infarction, which is consistent with our experimental results [57]. Nevertheless, some studies found that NGF pretreatment relieved myocardial infarct damage in the diabetic heart or physiologically normal heart by using a Langendorff system or in in vivo rat model of myocardial regional ischemia-reperfusion injury [58–60]. The interesting thing is that the results of these studies differ from our findings. The multiple functions of NGF make it difficult for researchers to study. The findings of our study indicate that overexpression of NGF exacerbates myocardial injury and neural remodeling. YQHX can also increase the secretion of Sema-3A and alleviate the excessive regeneration and remodeling of sympathetic nerves, which plays an important role in stabilizing the electrophysiological function of the infarct marginal area, reducing the occurrence of malignant arrhythmia after myocardial infarction, and alleviating ventricular remodeling. The experimental results also showed that although Sema-3A had the effect of antagonizing nerve remodeling, its effect on other factors was weak, still insufficient to resist the occurrence and development of nerve remodeling in the chronic phase. Compared with metoprolol, YQHX has better curative effect and stable effect in the chronic phase and can give full play to the advantages of multiple components, multiple pathways, and multiple targets to protect myocardium and improve cardiac function.

NPY is a peptide composed of 36 amino acids, which is widely present in the central and peripheral nervous systems [26]. NPY is the most abundant neuropeptide in the heart, found in sympathetic nerve cells, endocardium and cardiomyocytes, cardiac ganglia, and parasympathetic nerve cells. In the case of sympathetic nerve pathology caused by myocardial infarction, left ventricular hypertrophy, and heart failure, NPY is released from the sympathetic nerve of the heart [61] with increasing level of the plasma NPY [62]. And more and more evidence shows that NPY can participate in the occurrence of myocardial hypertrophy through direct or indirect means [27]. After activation of the sympathetic nervous system, high concentrations of catecholamines and neuropeptides can cause cardiomyocyte hypertrophy, stimulate the increase of cardiac fibroblast synthesis, promote myocardial interstitial fibrosis, and further promote myocardial remodeling. As a nonadrenergic and noncholinergic neurotransmitter, NPY can well reflect the activity of sympathetic nerves and the dynamic changes of sympathetic neurotransmitter releasing after myocardial infarction. Consistent with the results of Xie et al. [63], NPY reduction alleviated myocardial hypertrophy.

By measuring the area, perimeter, and diameter of cardiomyocytes, we verified the hypertrophy of cardiomyocytes after myocardial infarction and the improvement effect of YQHX. Cardiomyocyte hypertrophy can promote the release of atrial natriuretic peptide (ANP) in large quantities. Clinically, it can predict the left ventricular dysfunction during acute myocardial infarction. The elevated plasma ANP concentration at the subacute phase of AMI patients indicates a poor long-term prognosis. By observing the protein expression of ANP and *β*-MHC, we found that the changes in myocardial hypertrophy and neural remodeling are consistent. YQHX has a definite therapeutic effect on inhibiting myocardial cell hypertrophy.

Many studies [64] have shown that the activation of ERK1/2 signaling pathway plays a harmful role in myocardial remodeling, inflammation, apoptosis, oxidative stress, and other processes and is closely related to myocardial hypertrophy [65, 66]. The ERK1/2 signaling pathway involves in cell proliferation, transformation, and differentiation and has complex protective effects on cardiomyocytes and neuronal cells. ERK1/2 pathway may promote sympathetic nerve excitation through at least two mechanisms. P-ERK1/2 can activate a variety of nuclear transcription factors. Through this mechanism, ERK1/2 pathway can make the central excitatory neurochemical environment drive sympathetic activity in heart failure [67]. As a downstream protein of the ERK1/2 pathway, c-fos protein can regulate cardiac function and cardiac hypertrophy [68] while c-Myc is a driving factor for cardiac hypertrophy [69]. In order to clarify the mechanism of YQHX inhibiting myocardial hypertrophy, this experiment explored the expression of ERK1/2 and p-ERK1/2 proteins through *in vitro* and *in vivo* studies. We found through experiments that the qi-invigorating and blood-activating herbs and inhibitors can significantly downregulate the expression of p-ERK1/2, reduce the level of c-fos and c-Myc proteins, and protect the heart. Therefore, we speculated that YQHX may regulate the neural remodeling after myocardial infarction through the ERK1/2 signaling pathway and slow down the development of myocardial hypertrophy, but its specific mechanism still needs to be further studied.

## 5. Conclusion

To sum up, through the results of these experiments, we determined that YQHX can regulate the expression of nerve related factors after myocardial infarction and reduce myocardial hypertrophy. The mechanism is related to the ERK1/2 signaling pathway. However, the relationship between cardiac hypertrophy and neural remodeling and the regulatory mechanism of YQHX still need to be further studied by animal and cell experiments. With the in-depth research on sympathetic nerve remodeling after myocardial infarction, early intervention of neural remodeling related factors and the use of traditional Chinese medicine in the chronic phase may be an important target and treatment plan for the prevention and treatment of cardiovascular disease in the future.

## Data Availability

The data used to support the findings of this study are available from the corresponding author upon request.
